# Tris(2-benzamido­eth­yl)ammonium tetra­fluoro­borate

**DOI:** 10.1107/S1600536810024323

**Published:** 2010-06-26

**Authors:** Marcy L. Pilate, Henry Blount, Frank R. Fronczek, Md. Alamgir Hossain

**Affiliations:** a Department of Chemistry and Biochemistry, Jackson State University, Jackson, MS 39217, USA; b Department of Chemistry, Louisiana State University, Baton Rouge, LA 70803, USA

## Abstract

In the title compound, C_27_H_31_N_4_O_3_^+^·BF_4_^−^, the central N atom is protonated. The three arms form a pocket and one amidic O atom accepts an inter­molecular hydrogen bond with the protonated amine. The tetra­fluoro­borate anion is outside the cavity and is hydrogen bonded to one amide N atom. Adjacent organic cations are connected by a pair of N—H⋯O hydrogen bonds, forming a chain.

## Related literature

For general background to tris­(amino­eth­yl)–amine and its binding of anions, see: Bianchi *et al.* (1997 ▶); Kang *et al.* (2005 ▶); Hossain,(2008 ▶); For related structures, see: Bazzicalupi *et al.* (2009 ▶); Hossain *et al.* (2004 ▶); Burgess *et al.* (1991 ▶), Lo & Ng (2008 ▶); Saeed *et al.* (2010 ▶).
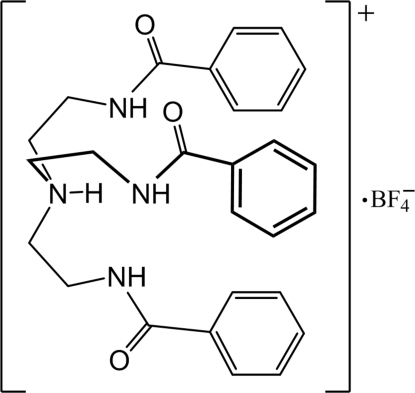


## Experimental

### 

#### Crystal data

C_27_H_31_N_4_O_3_^+^·BF_4_^−^
*M* *_r_* = 546.37
Monoclinic, 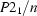

*a* = 13.325 (2) Å
*b* = 9.572 (2) Å
*c* = 21.118 (3) Å
β = 94.546 (10)°
*V* = 2685.1 (8) Å^3^
*Z* = 4
Mo *K*α radiation
μ = 0.11 mm^−1^
*T* = 90 K
0.27 × 0.25 × 0.10 mm

#### Data collection

Nonius KappaCCD diffractometer with Oxford Cryostream
31844 measured reflections
5914 independent reflections
3310 reflections with *I* > 2σ(*I*)
*R*_int_ = 0.068

#### Refinement

*R*[*F* ^2^ > 2σ(*F* ^2^)] = 0.048
*wR*(*F* ^2^) = 0.114
*S* = 1.00
5914 reflections
365 parameters
H atoms treated by a mixture of independent and constrained refinement
Δρ_max_ = 0.26 e Å^−3^
Δρ_min_ = −0.27 e Å^−3^

### 

Data collection: *COLLECT* (Nonius, 1999 ▶); cell refinement: *DENZO* and *SCALEPACK* (Otwinowski & Minor, 1997 ▶); data reduction: *DENZO* and *SCALEPACK*; program(s) used to solve structure: *SIR97* (Altomare *et al.*, 1999 ▶); program(s) used to refine structure: *SHELXL97* (Sheldrick, 2008 ▶); molecular graphics: *ORTEP-3* (Farrugia, 1997 ▶); software used to prepare material for publication: *SHELXL97*.

## Supplementary Material

Crystal structure: contains datablocks global, I. DOI: 10.1107/S1600536810024323/rk2214sup1.cif

Structure factors: contains datablocks I. DOI: 10.1107/S1600536810024323/rk2214Isup2.hkl

Additional supplementary materials:  crystallographic information; 3D view; checkCIF report

## Figures and Tables

**Table 1:** Hydrogen-bond geometry (Å, °)

*D*—H⋯*A*	*D*—H	H⋯*A*	*D*⋯*A*	*D*—H⋯*A*
N1—H1*N*⋯O1	1.003 (19)	1.70 (2)	2.646 (2)	155.2 (16)
N2—H2*N*⋯O3^i^	0.87 (2)	2.01 (2)	2.861 (2)	168.8 (19)
N3—H3*N*⋯F3	0.92 (2)	2.00 (2)	2.901 (2)	170.0 (18)
N4—H4*N*⋯O2^ii^	0.87 (2)	2.12 (2)	2.894 (2)	147.5 (19)

Symmetry codes: (i) 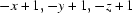; (ii) 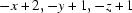.

**Fig. 1.**
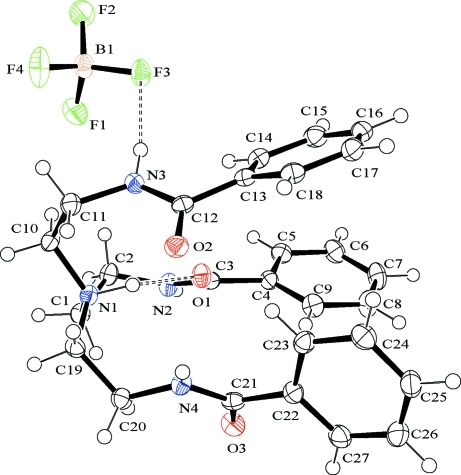
The molecular structure of the title compound with the atom numbering scheme. Displacement ellipsoids are drawn at the 50% probability level. H atoms are presented as a small spheres of arbitrary radius. Hydrogen bonds are presented by dashed lines.

**Fig. 2.**
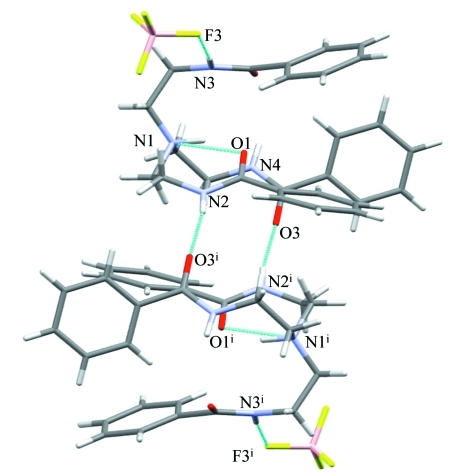
A view of the hydrogen bonded centrosymmetric dimer.

## supplementary crystallographic information

### Comment

Tris(aminoethyl)–amine, trend with *C3* symmetry is known to bind an anion (Burgess *et al.*, 1991; Hossain *et al.*, 2004; Bazzicalupi *et al.*,  2009) and is often used to build azacryptands with amine or amide functionality (Hossain, 2008). These molecules are of particularly interest for binding anions with three fold rotation axes, such as nitrate, phosphate, perchlorate and sulfate (Bianchi *et al.*, 1997). The binding is predominantly occurred through hydrogen bonding and electrostatic interactions of protonated amines or amidic protons with negatively charged species. For example, tetrahedral sulfate was seen to be encapsulated in amine cryptand or amide cryptand where the same trend units are as used as building blocks (Kang *et al.*, 2005). Herein, we report the molecularcv and crystal structures of the title compound in which a tetrafluoroborate anion is held outside the cavity with one hydrogen bond and remains outside the cavity. Single crystal analysis of the title compound reveals that the molecule crystallizes in its monoclinic space group forming a cavity with the three arms that are connected with one central amine (Fig. 1). The central amine is protonated and the chrage is balanced with one tetrafluoroborate that is located outside the cavity and is bonded with one amide nitrogen. In the single–crystal, one amide H points toward the cavity and makes an intermolecular hydrogen bond with the proton on the central amine with N···O distance of 2.864 (2)Å (Table 1). Two adjacent molecules are connected each other forming a centrosymmetric dimer with two hydrogen bonds with N···O bonds distances of 2.861 (2)Å and 2.894 (2)Å (Fig. 2). Similar intramolecular bonding was previously reported for mono–functional (Lo & Ng, 2008) or di–functional (Saeed *et al.*, 2010).

### Experimental

To a solution of tris(2–benzamidoethyl) amine (50 mg) in methanol (1 ml) was added a few drops fluoroboric acid at room temperature. The white powder formed was collected by filtration. Yield: 80%. M.P. 423 K. ^1^H NMR (500 MHz, D_2_O, *TSP*): *δ* 8.78 (b, 3H, NHCO), 7.81(d, 3H, *Ar*H), 7.72 (b, 1H, NHCH_2_), 7.56 (t, 3H, *Ar*H), 7.47 (t, 3H, *Ar*H), 3.72 (t, 6H, CH_2_NHCO), 3.35 (t, 6H, NHCH_2_). ^13^C NMR (125 MHz, D_2_O, *TSP*): *δ* 167.8 (CO), 138.9 (*Ar*C), 131.9 (*Ar*C), 128.8 (*Ar*C). 127.6 (*Ar*C), 52.4 (NHCH_2_), 34.8 (*C*H_2_CO). The salt was redissolved in water and crystals suitable for X–ray analysis were grown from slow evaporation of the solvent at room temperature.

### Refinement

H atoms on C were placed in idealized positions with C—H distances 0.95–0.99Å and thereafter treated as riding. The coordinates of those on N were refined. The *U*_iso_ for H was assigned as 1.2 times *U*_eq_ of the attached atom. The largest residual density peak was 1.50 Å from O2.

### Crystal data

**Table:**

C_27_H_31_N_4_O_3_^+^·BF_4_^−^	*F*(000) = 1144
*M**_r_* = 546.37	*D*_x_ = 1.352 Mg m^−^^3^
Monoclinic, *P*2_1_/*n*	Melting point: 423 K
Hall symbol: -P 2yn	Mo *K*α radiation, λ = 0.71073 Å
*a* = 13.325 (2) Å	Cell parameters from 6170 reflections
*b* = 9.572 (2) Å	θ = 2.5–27.1°
*c* = 21.118 (3) Å	µ = 0.11 mm^−^^1^
β = 94.546 (10)°	*T* = 90 K
*V* = 2685.1 (8) Å^3^	Block, colourless
*Z* = 4	0.27 × 0.25 × 0.10 mm

### Data collection

**Table:**

Nonius KappaCCD diffractometer with Oxford Cryostream	3310 reflections with *I* > 2σ(*I*)
Radiation source: fine–focus sealed tube	*R*_int_ = 0.068
graphite	θ_max_ = 27.1°, θ_min_ = 2.6°
ω–scans with κ–offsets	*h* = −17→17
31844 measured reflections	*k* = −12→12
5914 independent reflections	*l* = −27→26

### Refinement

**Table:**

Refinement on *F*^2^	Secondary atom site location: difference Fourier map
Least-squares matrix: full	Hydrogen site location: inferred from neighbouring sites
*R*[*F*^2^ > 2σ(*F*^2^)] = 0.048	H atoms treated by a mixture of independent and constrained refinement
*wR*(*F*^2^) = 0.114	*w* = 1/[σ^2^(*F*_o_^2^) + (0.0492*P*)^2^] where *P* = (*F*_o_^2^ + 2*F*_c_^2^)/3
*S* = 1.00	(Δ/σ)_max_ = 0.001
5914 reflections	Δρ_max_ = 0.26 e Å^−^^3^
365 parameters	Δρ_min_ = −0.27 e Å^−^^3^
0 restraints	Extinction correction: *SHELXL97* (Sheldrick, 2008), Fc^*^=kFc[1+0.001xFc^2^λ^3^/sin(2θ)]^-1/4^
Primary atom site location: structure-invariant direct methods	Extinction coefficient: 0.0047 (7)

### Special details

**Table:**

Geometry. All s.u.'s (except the s.u. in the dihedral angle between two l.s. planes) are estimated using the full covariance matrix. The cell s.u.'s are taken into account individually in the estimation of s.u.'s in distances, angles and torsion angles; correlations between s.u.'s in cell parameters are only used when they are defined by crystal symmetry. An approximate (isotropic) treatment of cell s.u.'s is used for estimating s.u.'s involving l.s. planes.
Refinement. Refinement of *F*^2^ against ALL reflections. The weighted *R*–factor *wR* and goodness of fit *S* are based on *F*^2^, conventional *R*–factors *R* are based on *F*, with *F* set to zero for negative *F*^2^. The threshold expression of *F*^2^ > σ(*F*^2^) is used only for calculating *R*–factors(gt) *etc*. and is not relevant to the choice of reflections for refinement. *R*–factors based on *F*^2^ are statistically about twice as large as those based on *F*, and *R*–factors based on ALL data will be even larger.

### Fractional atomic coordinates and isotropic or equivalent isotropic displacement parameters (Å^2^)

**Table:**

	*x*	*y*	*z*	*U*_iso_*/*U*_eq_	
O1	0.69598 (10)	0.66708 (15)	0.51248 (6)	0.0235 (3)	
O2	0.97004 (10)	0.65535 (14)	0.51217 (6)	0.0237 (3)	
O3	0.66033 (11)	0.35955 (15)	0.42976 (6)	0.0269 (4)	
N1	0.77743 (12)	0.52786 (18)	0.61191 (7)	0.0190 (4)	
H1N	0.7663 (14)	0.584 (2)	0.5718 (9)	0.023*	
N2	0.55317 (13)	0.65036 (19)	0.56356 (7)	0.0227 (4)	
H2N	0.4881 (16)	0.655 (2)	0.5611 (9)	0.027*	
N3	0.89693 (13)	0.78188 (18)	0.58685 (7)	0.0207 (4)	
H3N	0.8721 (15)	0.867 (2)	0.5972 (9)	0.025*	
N4	0.81500 (13)	0.39353 (18)	0.47972 (7)	0.0204 (4)	
H4N	0.8780 (16)	0.412 (2)	0.4753 (9)	0.024*	
C1	0.67293 (15)	0.4962 (2)	0.63108 (9)	0.0212 (5)	
H1A	0.6782	0.4611	0.6753	0.025*	
H1B	0.6432	0.4208	0.6035	0.025*	
C2	0.60230 (16)	0.6219 (2)	0.62667 (8)	0.0238 (5)	
H2A	0.5495	0.6073	0.6564	0.029*	
H2B	0.6412	0.7058	0.6411	0.029*	
C3	0.60327 (16)	0.6812 (2)	0.51281 (9)	0.0208 (5)	
C4	0.54443 (15)	0.7377 (2)	0.45532 (9)	0.0203 (5)	
C5	0.46519 (16)	0.8306 (2)	0.45962 (9)	0.0243 (5)	
H5	0.4442	0.8555	0.5001	0.029*	
C6	0.41677 (16)	0.8870 (2)	0.40503 (10)	0.0293 (5)	
H6	0.3632	0.9516	0.4081	0.035*	
C7	0.44618 (16)	0.8496 (2)	0.34635 (10)	0.0310 (6)	
H7	0.4121	0.8875	0.3090	0.037*	
C8	0.52504 (17)	0.7572 (2)	0.34138 (10)	0.0312 (6)	
H8	0.5447	0.7313	0.3008	0.037*	
C9	0.57503 (16)	0.7029 (2)	0.39571 (9)	0.0269 (5)	
H9	0.6305	0.6417	0.3924	0.032*	
C10	0.83608 (15)	0.6116 (2)	0.66272 (9)	0.0218 (5)	
H10A	0.8600	0.5483	0.6978	0.026*	
H10B	0.7908	0.6814	0.6801	0.026*	
C11	0.92603 (15)	0.6867 (2)	0.63859 (9)	0.0224 (5)	
H11A	0.9612	0.7398	0.6740	0.027*	
H11B	0.9737	0.6168	0.6239	0.027*	
C12	0.92266 (15)	0.7599 (2)	0.52720 (9)	0.0197 (5)	
C13	0.88929 (15)	0.8684 (2)	0.47918 (9)	0.0191 (5)	
C14	0.80176 (16)	0.9456 (2)	0.48281 (9)	0.0237 (5)	
H14	0.7618	0.9325	0.5176	0.028*	
C15	0.77232 (17)	1.0423 (2)	0.43572 (9)	0.0270 (5)	
H15	0.7120	1.0942	0.4382	0.032*	
C16	0.83094 (17)	1.0627 (2)	0.38534 (9)	0.0267 (5)	
H16	0.8112	1.1296	0.3535	0.032*	
C17	0.91824 (17)	0.9861 (2)	0.38106 (9)	0.0272 (5)	
H17	0.9583	1.0000	0.3463	0.033*	
C18	0.94687 (16)	0.8890 (2)	0.42784 (9)	0.0231 (5)	
H18	1.0065	0.8359	0.4248	0.028*	
C19	0.83279 (16)	0.3959 (2)	0.59740 (8)	0.0211 (5)	
H19A	0.8299	0.3309	0.6337	0.025*	
H19B	0.9045	0.4192	0.5935	0.025*	
C20	0.79230 (16)	0.3222 (2)	0.53764 (8)	0.0223 (5)	
H20A	0.8210	0.2268	0.5374	0.027*	
H20B	0.7184	0.3131	0.5381	0.027*	
C21	0.74826 (15)	0.4008 (2)	0.42910 (9)	0.0199 (5)	
C22	0.78649 (15)	0.4566 (2)	0.36920 (9)	0.0211 (5)	
C23	0.86024 (15)	0.5588 (2)	0.36906 (9)	0.0228 (5)	
H23	0.8870	0.5989	0.4080	0.027*	
C24	0.89490 (16)	0.6025 (2)	0.31219 (9)	0.0280 (5)	
H24	0.9440	0.6746	0.3122	0.034*	
C25	0.85853 (18)	0.5420 (2)	0.25530 (9)	0.0308 (6)	
H25	0.8844	0.5699	0.2166	0.037*	
C26	0.78424 (18)	0.4403 (2)	0.25513 (10)	0.0338 (6)	
H26	0.7589	0.3986	0.2163	0.041*	
C27	0.74720 (17)	0.4001 (2)	0.31173 (9)	0.0274 (5)	
H27	0.6945	0.3332	0.3114	0.033*	
B1	0.8258 (2)	1.0373 (3)	0.69733 (11)	0.0262 (6)	
F1	0.74417 (10)	0.95062 (13)	0.70473 (5)	0.0393 (4)	
F2	0.81014 (10)	1.16644 (13)	0.72363 (5)	0.0396 (4)	
F3	0.83701 (9)	1.05339 (12)	0.63193 (5)	0.0297 (3)	
F4	0.91193 (10)	0.97574 (15)	0.72626 (5)	0.0444 (4)	

### Atomic displacement parameters (Å^2^)

**Table:**

	*U*^11^	*U*^22^	*U*^33^	*U*^12^	*U*^13^	*U*^23^
O1	0.0192 (9)	0.0295 (9)	0.0217 (7)	0.0012 (7)	0.0004 (6)	0.0045 (6)
O2	0.0247 (9)	0.0212 (9)	0.0252 (8)	0.0040 (7)	0.0008 (6)	−0.0025 (6)
O3	0.0196 (9)	0.0279 (9)	0.0329 (8)	0.0000 (7)	0.0000 (6)	0.0030 (7)
N1	0.0182 (10)	0.0217 (10)	0.0169 (9)	−0.0006 (8)	−0.0003 (7)	0.0011 (7)
N2	0.0147 (10)	0.0320 (11)	0.0213 (9)	0.0018 (9)	0.0014 (8)	0.0021 (8)
N3	0.0235 (11)	0.0182 (10)	0.0201 (9)	0.0020 (8)	0.0010 (7)	0.0013 (8)
N4	0.0170 (10)	0.0250 (10)	0.0192 (9)	−0.0013 (9)	0.0013 (8)	−0.0006 (8)
C1	0.0182 (12)	0.0258 (13)	0.0199 (11)	−0.0029 (10)	0.0034 (8)	0.0007 (9)
C2	0.0230 (12)	0.0304 (13)	0.0181 (11)	0.0016 (10)	0.0027 (9)	0.0003 (9)
C3	0.0210 (13)	0.0188 (12)	0.0223 (11)	−0.0010 (10)	0.0007 (9)	−0.0017 (9)
C4	0.0183 (12)	0.0212 (12)	0.0209 (11)	−0.0012 (10)	−0.0010 (9)	0.0017 (9)
C5	0.0226 (13)	0.0241 (12)	0.0263 (12)	−0.0013 (10)	0.0036 (9)	0.0007 (10)
C6	0.0250 (13)	0.0293 (14)	0.0333 (13)	0.0044 (11)	0.0010 (10)	0.0069 (11)
C7	0.0247 (13)	0.0398 (15)	0.0275 (12)	0.0034 (12)	−0.0040 (10)	0.0123 (11)
C8	0.0321 (14)	0.0391 (15)	0.0224 (12)	0.0044 (12)	0.0024 (10)	0.0050 (10)
C9	0.0231 (13)	0.0292 (14)	0.0283 (12)	0.0022 (10)	0.0021 (9)	0.0023 (10)
C10	0.0237 (12)	0.0236 (12)	0.0173 (10)	−0.0001 (10)	−0.0028 (9)	−0.0019 (9)
C11	0.0212 (12)	0.0236 (12)	0.0218 (11)	0.0013 (10)	−0.0010 (9)	0.0010 (9)
C12	0.0172 (12)	0.0195 (12)	0.0221 (11)	−0.0051 (10)	−0.0012 (9)	−0.0019 (9)
C13	0.0185 (12)	0.0173 (12)	0.0212 (11)	−0.0019 (10)	−0.0004 (8)	−0.0035 (9)
C14	0.0274 (13)	0.0228 (12)	0.0206 (11)	−0.0009 (11)	0.0010 (9)	−0.0039 (10)
C15	0.0284 (13)	0.0226 (13)	0.0292 (12)	0.0035 (11)	−0.0024 (10)	−0.0026 (10)
C16	0.0325 (14)	0.0207 (12)	0.0253 (12)	−0.0028 (11)	−0.0065 (10)	0.0017 (10)
C17	0.0302 (14)	0.0286 (14)	0.0225 (12)	−0.0068 (11)	0.0009 (9)	−0.0002 (10)
C18	0.0206 (12)	0.0225 (12)	0.0261 (11)	−0.0021 (10)	0.0016 (9)	−0.0034 (10)
C19	0.0239 (12)	0.0181 (12)	0.0213 (11)	0.0051 (10)	0.0019 (9)	0.0012 (9)
C20	0.0248 (13)	0.0215 (12)	0.0210 (11)	0.0003 (10)	0.0048 (9)	0.0006 (9)
C21	0.0159 (12)	0.0168 (11)	0.0268 (12)	0.0024 (10)	0.0004 (9)	−0.0039 (9)
C22	0.0184 (12)	0.0216 (12)	0.0225 (11)	0.0055 (10)	−0.0029 (9)	0.0014 (9)
C23	0.0243 (13)	0.0236 (13)	0.0203 (11)	0.0040 (10)	0.0012 (9)	−0.0021 (9)
C24	0.0264 (13)	0.0264 (13)	0.0312 (13)	0.0020 (11)	0.0021 (10)	0.0031 (10)
C25	0.0428 (16)	0.0291 (14)	0.0209 (12)	0.0080 (12)	0.0050 (10)	0.0042 (10)
C26	0.0472 (16)	0.0296 (14)	0.0231 (12)	0.0057 (13)	−0.0079 (11)	0.0007 (10)
C27	0.0294 (14)	0.0230 (13)	0.0285 (12)	−0.0003 (11)	−0.0058 (10)	0.0036 (10)
B1	0.0297 (16)	0.0295 (16)	0.0199 (13)	0.0049 (13)	0.0043 (11)	0.0011 (11)
F1	0.0437 (9)	0.0353 (8)	0.0392 (7)	−0.0023 (7)	0.0056 (6)	0.0090 (6)
F2	0.0628 (10)	0.0305 (8)	0.0269 (7)	0.0042 (7)	0.0127 (6)	−0.0040 (6)
F3	0.0397 (8)	0.0307 (8)	0.0194 (6)	0.0108 (6)	0.0058 (5)	0.0022 (5)
F4	0.0383 (9)	0.0703 (11)	0.0249 (7)	0.0223 (8)	0.0048 (6)	0.0119 (7)

### Geometric parameters (Å, °)

**Table:**

O1—C3	1.243 (2)	C11—H11A	0.9900
O2—C12	1.238 (2)	C11—H11B	0.9900
O3—C21	1.238 (2)	C12—C13	1.495 (3)
N1—C19	1.506 (2)	C13—C14	1.388 (3)
N1—C10	1.507 (2)	C13—C18	1.391 (3)
N1—C1	1.511 (2)	C14—C15	1.392 (3)
N1—H1N	1.003 (19)	C14—H14	0.9500
N2—C3	1.340 (2)	C15—C16	1.383 (3)
N2—C2	1.463 (2)	C15—H15	0.9500
N2—H2N	0.87 (2)	C16—C17	1.384 (3)
N3—C12	1.348 (2)	C16—H16	0.9500
N3—C11	1.452 (2)	C17—C18	1.388 (3)
N3—H3N	0.92 (2)	C17—H17	0.9500
N4—C21	1.337 (2)	C18—H18	0.9500
N4—C20	1.453 (2)	C19—C20	1.508 (3)
N4—H4N	0.87 (2)	C19—H19A	0.9900
C1—C2	1.526 (3)	C19—H19B	0.9900
C1—H1A	0.9900	C20—H20A	0.9900
C1—H1B	0.9900	C20—H20B	0.9900
C2—H2A	0.9900	C21—C22	1.499 (3)
C2—H2B	0.9900	C22—C23	1.387 (3)
C3—C4	1.493 (3)	C22—C27	1.392 (3)
C4—C5	1.389 (3)	C23—C24	1.385 (3)
C4—C9	1.394 (3)	C23—H23	0.9500
C5—C6	1.385 (3)	C24—C25	1.386 (3)
C5—H5	0.9500	C24—H24	0.9500
C6—C7	1.376 (3)	C25—C26	1.388 (3)
C6—H6	0.9500	C25—H25	0.9500
C7—C8	1.384 (3)	C26—C27	1.384 (3)
C7—H7	0.9500	C26—H26	0.9500
C8—C9	1.382 (3)	C27—H27	0.9500
C8—H8	0.9500	B1—F2	1.378 (3)
C9—H9	0.9500	B1—F1	1.387 (3)
C10—C11	1.520 (3)	B1—F4	1.388 (3)
C10—H10A	0.9900	B1—F3	1.409 (2)
C10—H10B	0.9900		
			
C19—N1—C10	110.86 (15)	O2—C12—N3	122.60 (18)
C19—N1—C1	111.27 (16)	O2—C12—C13	121.20 (17)
C10—N1—C1	110.66 (15)	N3—C12—C13	116.19 (18)
C19—N1—H1N	108.6 (11)	C14—C13—C18	119.04 (19)
C10—N1—H1N	110.5 (11)	C14—C13—C12	122.60 (17)
C1—N1—H1N	104.8 (11)	C18—C13—C12	118.33 (18)
C3—N2—C2	123.66 (18)	C13—C14—C15	120.34 (19)
C3—N2—H2N	120.1 (13)	C13—C14—H14	119.8
C2—N2—H2N	115.9 (13)	C15—C14—H14	119.8
C12—N3—C11	122.19 (18)	C16—C15—C14	119.9 (2)
C12—N3—H3N	119.1 (12)	C16—C15—H15	120.0
C11—N3—H3N	117.5 (12)	C14—C15—H15	120.0
C21—N4—C20	121.86 (18)	C15—C16—C17	120.3 (2)
C21—N4—H4N	119.3 (13)	C15—C16—H16	119.9
C20—N4—H4N	116.9 (13)	C17—C16—H16	119.9
N1—C1—C2	113.71 (17)	C16—C17—C18	119.6 (2)
N1—C1—H1A	108.8	C16—C17—H17	120.2
C2—C1—H1A	108.8	C18—C17—H17	120.2
N1—C1—H1B	108.8	C17—C18—C13	120.8 (2)
C2—C1—H1B	108.8	C17—C18—H18	119.6
H1A—C1—H1B	107.7	C13—C18—H18	119.6
N2—C2—C1	115.56 (16)	N1—C19—C20	114.47 (16)
N2—C2—H2A	108.4	N1—C19—H19A	108.6
C1—C2—H2A	108.4	C20—C19—H19A	108.6
N2—C2—H2B	108.4	N1—C19—H19B	108.6
C1—C2—H2B	108.4	C20—C19—H19B	108.6
H2A—C2—H2B	107.5	H19A—C19—H19B	107.6
O1—C3—N2	122.62 (18)	N4—C20—C19	113.57 (17)
O1—C3—C4	119.51 (17)	N4—C20—H20A	108.9
N2—C3—C4	117.86 (18)	C19—C20—H20A	108.9
C5—C4—C9	119.34 (18)	N4—C20—H20B	108.9
C5—C4—C3	122.10 (17)	C19—C20—H20B	108.9
C9—C4—C3	118.41 (19)	H20A—C20—H20B	107.7
C6—C5—C4	120.12 (19)	O3—C21—N4	122.99 (18)
C6—C5—H5	119.9	O3—C21—C22	120.58 (17)
C4—C5—H5	119.9	N4—C21—C22	116.38 (18)
C7—C6—C5	120.0 (2)	C23—C22—C27	119.33 (19)
C7—C6—H6	120.0	C23—C22—C21	122.77 (17)
C5—C6—H6	120.0	C27—C22—C21	117.88 (19)
C6—C7—C8	120.5 (2)	C24—C23—C22	119.99 (19)
C6—C7—H7	119.8	C24—C23—H23	120.0
C8—C7—H7	119.8	C22—C23—H23	120.0
C9—C8—C7	119.8 (2)	C23—C24—C25	120.5 (2)
C9—C8—H8	120.1	C23—C24—H24	119.8
C7—C8—H8	120.1	C25—C24—H24	119.8
C8—C9—C4	120.3 (2)	C24—C25—C26	119.7 (2)
C8—C9—H9	119.9	C24—C25—H25	120.1
C4—C9—H9	119.9	C26—C25—H25	120.1
N1—C10—C11	113.03 (15)	C27—C26—C25	119.7 (2)
N1—C10—H10A	109.0	C27—C26—H26	120.1
C11—C10—H10A	109.0	C25—C26—H26	120.1
N1—C10—H10B	109.0	C26—C27—C22	120.6 (2)
C11—C10—H10B	109.0	C26—C27—H27	119.7
H10A—C10—H10B	107.8	C22—C27—H27	119.7
N3—C11—C10	112.24 (16)	F2—B1—F1	110.24 (19)
N3—C11—H11A	109.2	F2—B1—F4	110.49 (19)
C10—C11—H11A	109.2	F1—B1—F4	108.96 (19)
N3—C11—H11B	109.2	F2—B1—F3	109.16 (19)
C10—C11—H11B	109.2	F1—B1—F3	108.70 (18)
H11A—C11—H11B	107.9	F4—B1—F3	109.26 (18)
			
C19—N1—C1—C2	−162.24 (15)	N3—C12—C13—C18	151.42 (18)
C10—N1—C1—C2	74.0 (2)	C18—C13—C14—C15	−0.1 (3)
C3—N2—C2—C1	−61.3 (3)	C12—C13—C14—C15	−177.89 (18)
N1—C1—C2—N2	83.1 (2)	C13—C14—C15—C16	−0.7 (3)
C2—N2—C3—O1	11.6 (3)	C14—C15—C16—C17	0.8 (3)
C2—N2—C3—C4	−167.04 (19)	C15—C16—C17—C18	−0.3 (3)
O1—C3—C4—C5	−140.5 (2)	C16—C17—C18—C13	−0.5 (3)
N2—C3—C4—C5	38.1 (3)	C14—C13—C18—C17	0.6 (3)
O1—C3—C4—C9	35.0 (3)	C12—C13—C18—C17	178.56 (18)
N2—C3—C4—C9	−146.4 (2)	C10—N1—C19—C20	−166.83 (16)
C9—C4—C5—C6	0.5 (3)	C1—N1—C19—C20	69.6 (2)
C3—C4—C5—C6	175.96 (19)	C21—N4—C20—C19	−140.97 (19)
C4—C5—C6—C7	0.9 (3)	N1—C19—C20—N4	72.5 (2)
C5—C6—C7—C8	−1.0 (3)	C20—N4—C21—O3	6.9 (3)
C6—C7—C8—C9	−0.4 (4)	C20—N4—C21—C22	−170.42 (17)
C7—C8—C9—C4	1.9 (3)	O3—C21—C22—C23	149.1 (2)
C5—C4—C9—C8	−1.9 (3)	N4—C21—C22—C23	−33.5 (3)
C3—C4—C9—C8	−177.53 (19)	O3—C21—C22—C27	−32.3 (3)
C19—N1—C10—C11	75.0 (2)	N4—C21—C22—C27	145.04 (19)
C1—N1—C10—C11	−161.06 (17)	C27—C22—C23—C24	−0.8 (3)
C12—N3—C11—C10	−112.8 (2)	C21—C22—C23—C24	177.74 (19)
N1—C10—C11—N3	58.7 (2)	C22—C23—C24—C25	−1.9 (3)
C11—N3—C12—O2	2.2 (3)	C23—C24—C25—C26	2.3 (3)
C11—N3—C12—C13	−178.72 (17)	C24—C25—C26—C27	−0.1 (3)
O2—C12—C13—C14	148.4 (2)	C25—C26—C27—C22	−2.5 (3)
N3—C12—C13—C14	−30.7 (3)	C23—C22—C27—C26	3.0 (3)
O2—C12—C13—C18	−29.5 (3)	C21—C22—C27—C26	−175.61 (19)

### Hydrogen-bond geometry (Å, °)

**Table:**

*D*—H···*A*	*D*—H	H···*A*	*D*···*A*	*D*—H···*A*
N1—H1N···O1	1.003 (19)	1.70 (2)	2.646 (2)	155.2 (16)
N2—H2N···O3^i^	0.87 (2)	2.01 (2)	2.861 (2)	168.8 (19)
N3—H3N···F3	0.92 (2)	2.00 (2)	2.901 (2)	170.0 (18)
N4—H4N···O2^ii^	0.87 (2)	2.12 (2)	2.894 (2)	147.5 (19)

Symmetry codes: (i) −*x*+1, −*y*+1, −*z*+1; (ii) −*x*+2, −*y*+1, −*z*+1.
